# Postoperative clinical outcome and complications of combined cannulated cancellous screw with Kirschner wire in adolescent femoral neck fractures

**DOI:** 10.3389/fped.2023.1169581

**Published:** 2023-05-16

**Authors:** Guy Romeo Kenmegne, Chang Zou, Yixiang Lin, Yijie Yin, Shengbo Huang, Yue Fang

**Affiliations:** Department of Orthopaedics, West China Hospital, West China School of Medicine, Sichuan University, Chengdu, China

**Keywords:** adolescent, avascular necrosis, transphyseal fixation, femoral neck fractures, Delbet classification

## Abstract

**Purpose:**

Fractures of the femoral neck account for less than 1% of pediatric and adolescent fractures. Due to the high incidence of complications, and the age of the patients, the choice of fixation approach remains controversial among orthopedic surgeons. This study aimed to evaluate the postoperative outcomes and complications of femoral neck fracture in adolescents with open physis, following transphyseal fixation using a combined cannulated cancellous screw and Kirschner wire fixation.

**Methods:**

Data of 19 patients aged between 12 and 19 years from January 2010 to January 2021 were retrospectively studied. The follow-up period was 1–11 years (5.83 ± 3.76 years). The variables of interest including demographic and clinical variables [age, BMI, gender, side of injury, fracture classification, operation time, time to surgery, and length of hospital stay (LOS)], postoperative outcomes, and complications (fracture healing time, nonunion, coxa vara, osteoarthritis, avascular necrosis, screw loosening, and femoral shortening) were analyzed. The assessment of the hip function was done on the final follow-up using the Ratliff scoring system.

**Results:**

There was a male predominance of 76%; the mean age was 16.14 ± 1.57 years and the most frequent mechanism of injury was fall from a height. Delbet type II and III were the most encountered. The mean intraoperative time was 54.71 ± 7.85 min, the LOS was 8.34 ± 1.81days, and the time to surgery was 2.60 ± 1.16 days; the fracture healing time was 3.31 ± 1.04 months. The postoperative complications encountered were coxa vara osteoarthritis, spontaneous dislocation, and neck shortening. Clinical assessment revealed good results in 89% of patients and fair results in 11% of patients.

**Conclusion:**

Transphyseal fixation using cannulated cancellous screw combined with Kirschner wire in our patients provided acceptable results. Thus, this approach can be a viable alternative in the management of adolescent femoral neck fracture with open physis.

## 1. Introduction

Fractures of the femoral neck in the pediatric population are rare and account for less than 1% of pediatric and adolescent fractures (1–3). The femoral neck is covered with a thick and strong periosteum in children/adolescents; as a result, 80–90% of fractures affecting the femoral neck in this group are related to high energy trauma (high-force impact) (1). These fractures are usually described in patients who sustained femoral neck fracture during sport activities. According to the literature, 11% of adolescent femoral neck fracture occurs during sport activity; they are commonly found in athletes and military recruits at a rate of 5% (4, 5). Although it is very rare, they are potentially one of the most serious injuries (6, 7). They carry an important risk of osteoarthritis, fracture nonunion, and femoral neck avascular necrosis (AVN) (5). There is an absence of vascular anastomosis between the femoral neck in children; as a result, the incidence of avascular necrosis after displaced femoral neck fracture is higher in this population group than in adults (8–10).

Historically, different management options for femoral neck fractures in pediatric patients have been of routine use including conservative management with prolonged balanced traction, prolonged casting, percutaneous fixation, closed reduction-internal fixation, and open reduction and fixation with K-wires (11–14). The primary treatment goal is to achieve accurate anatomical reduction and stable internal fixation, thus, avoiding fracture complications, but the best treatment approach remained controversial (15). Recently, some scholars recommended the use of 6.5 or 7.3 mm cannulated cancellous screws (CCSs) in Delbet type I–III adolescent hip fractures (in patients aged above 10 years) (16). The stress fracture affecting the adolescent femoral neck could be as the result of bone fragility (low bone mineral density, BMD) and bone size (smaller diameter of pediatric femoral neck). Adequate bone density is achieved in individuals in late adolescent age (11–13 years in females and 13–17 years in males) (17).

We hypothesized that lower bone size, density, and vascular vulnerability could affect the outcome after the fracture fixation and lead to several complications, especially avascular necrosis. Therefore, we thought that a transphyseal fixation combining multiple cannulated screws with 2.5 mm Ø Kirschner wire could increase the strength of the internal fixation, reducing the risk of fixation failure and the incidence of avascular necrosis in adolescent femoral neck fracture. The aim of this study was to present the postoperative outcomes of femoral neck fracture in adolescents with open physis, following transphyseal fixation with combined cannulated cancellous screws and Kirschner wire.

## 2 Patients and methods

### 2.1. Patients

We retrospectively studied all adolescent individuals aged 12–19 years admitted at the first-level trauma center of our institution with femoral neck fracture from January 2011 to January 2021. First, we selected 43 patients with femoral neck fracture (based on plain radiographs and 3D CT scan images), classified according to the Delbet system as transepiphyseal (type I), transcervical (type II), cervicotrochanteric (type III), and intertrochanteric (type IV).

The inclusion criteria were: individuals of adolescent age (12–19 years) (18, 19), patients with displaced fractures, patients with open physis, fractures managed surgically exclusively with transphyseal fixation, and patients managed by the same medical team. The exclusion criteria were: patients with closed physis, fractures managed conservatively, patients with some metabolic bone diseases, patients with pathologic fractures, patients with less than 1 year follow-up, and patients with other associated injuries.

Overall, 19 out of 43 patients met the inclusion criteria and were enrolled in this study. Patients’ data, including postoperative outcome, were collected from medical records, especially the information recorded during the outpatient clinic follow-up visit and the imaging assessment done with standard x-Ray films; those with complains of pain around the affected hip were recommended an MRI to exclude AVN. Data were collected following the anonymous method, and personal identity was completely erased in the data collecting form for ethical consideration. Informed consent was obtained from the parents of all the patients included in this study by phone call using the number found in the medical record, and the study was approved by the institutional review board and the ethics committee of our institution.

### 2.2. Classification and treatment plan

The Delbet classification was used to evaluate the risk of complication and was categorized following the definition available in the literature as Delbet type I (transepiphyseal), type II (transcervical), type III (cervicotrochanteric), and type IV (intertrochanteric) (7, 8, 10, 20).

Patients who were included in this study were classified as Delbet types II–IV and were managed operatively. The cross-epiphyseal (transepiphyseal) CCS combined with Kirschner wire was the standard approach technique.

### 2.3. Surgical technique

**Figure 1:**
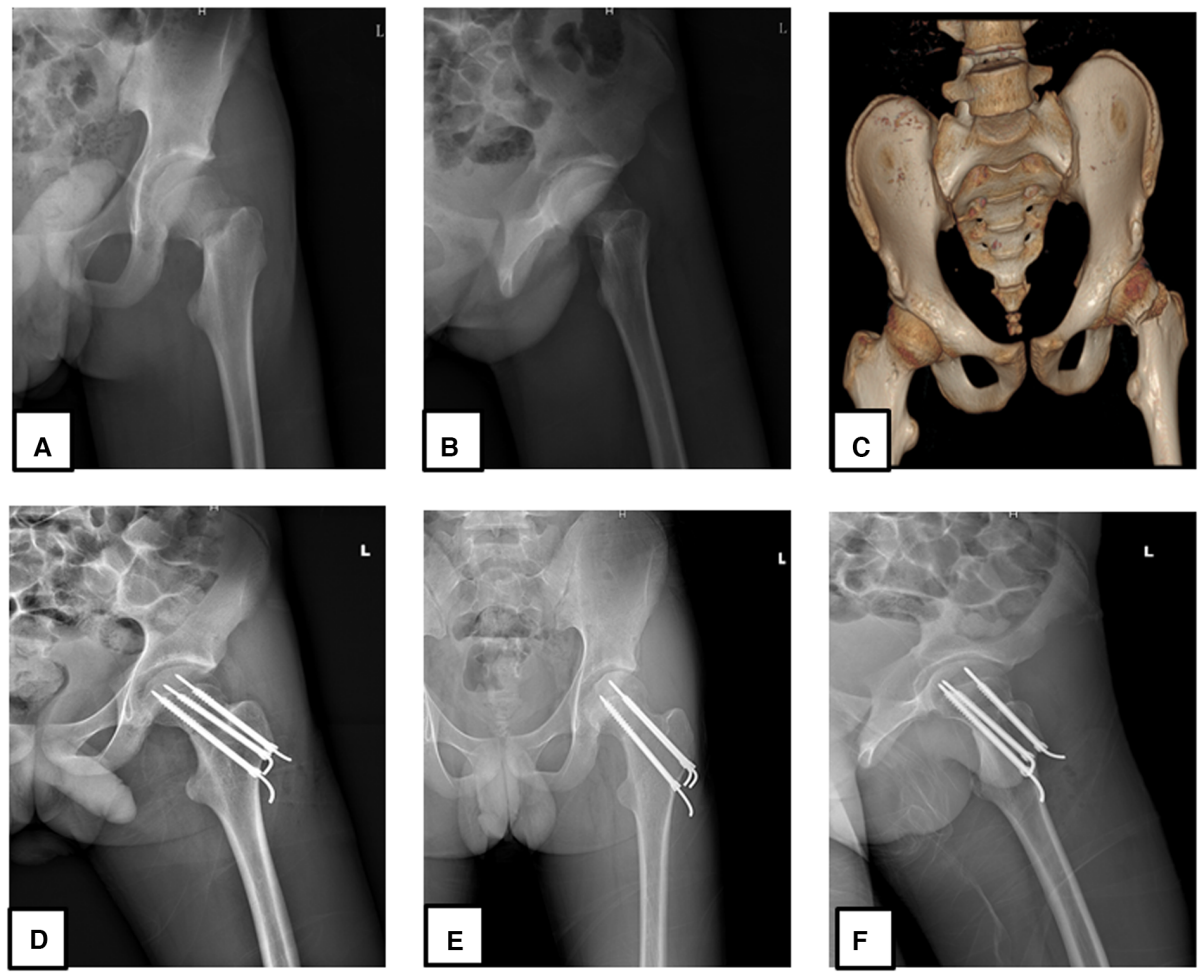
A 14-year-old male patient who sustained a Delbet type II femoral neck fracture after a fall, treated with cannulated cancellous screw combined with K-wire 24 h after injury. Immediate preoperative x-ray anteroposterior view (**A**), lateral view (**B**), 3D CT scan anteroposterior view (**C**), immediate postoperative x-ray anteroposterior view (**D,F**), and lateral view (**E**) were done.

Under the traction frame, femoral neck fractures were reduced using a combination of mechanisms as follows: traction-adduction and internal rotation. When the reduction was satisfied in the anteroposterior and lateral fluoroscopic views, the patient's skin preparation was done with a strict aseptic protocol. An incision of about 3–5 cm in length was made on the lateral side of the hip. The stepwise dissection was done starting from the skin, subcutaneous fascia, deep fascia, vastus lateralis muscle, and the periosteum. The pins guide wire (Kirschner wire) was positioned under fluoroscopic guidance within the femoral neck through the head. The three guide pins were placed in an inverted triangular design with the apex oriented downward. After confirming the correct positions of the three guide wires under fluoroscopy, the lateral cortex was drilled, and three cannulated screws were inserted. The femoral neck was completely reduced and the reduction was satisfactory on the fluoroscopic radiographs. The Kirschner wires were left within the cannulated screws (across the epiphysis) to strengthen the internal fixation (Figures 1C–F). The outer extremity of the guide wire was shortened to a suitable length (Figure 1D) and left within the cannulated screw.

### 2.4. Postoperative protocol

To all patients, a routine outpatient clinic follow-up was recommended weekly for the first month, twice the following month, and then once every 3 months afterward until removal of the internal fixation. The postoperative regular radiographs (anteroposterior and lateral view) were done on every outpatient visit to evaluate the clinical outcome such as fracture healing and potential complications (AVN, arthritis, shortening, coxa vara). The postoperative management guideline consisted of non-weight-bearing period of 4 weeks followed by partial weight-bearing for an additional 6 weeks.

After complete healing was noticed on the radiograph, we proceeded with partial removal of the internal fixation (the three supportive Kirschner wires with two CCS) usually around 6 months after the operation depending on the clinical appreciation on the radiographs. Complete removal was done after the following three months. This sequential removal allows a complete consolidation and full healing of the fractured neck before complete nail removal is done.

### 2.5. Assessment

We analyzed, assessed, and recorded all clinical data and outcomes of the patients considered in this study from medical record. We recorded in the data collecting form all the demographic variables of the patients and postoperative follow-up variables such as (1) follow-up period, (2) fracture healing time, (3) nonunion rate, (4) coxa varus rate, (5) osteoarthritis rate, (6) femoral neck avascular necrosis rate, (7) aseptic screw loosening rate, (8) femoral neck shortening rate, and (9) clinical assessment using the Ratliff scoring system [using the criteria of pain tolerance, range of motion/movement (ROM), level of tolerance to physical activities, and radiological assessment] at the last follow-up (Table 1) (6). The follow-up radiographs were used to determine the time to union, avascular necrosis, and the presence of posttraumatic osteoarthritis, and the results were found in the medical records. The extent of femoral neck shortening was measured using standard anteroposterior radiographs and was classified into three categories: none or mild (<0.5 cm), moderate (0.5–1 cm), and severe (>1 cm).

**Table 1:** Ratliff's criteria.

	Good	Fair	Poor
Pain	None	Occasional	Disabling
Movement	Full or minimal restriction	>50%	<50%
Activity	Normal	Avoids games	Restricted
Radiological assessment	Normal or mild deformity	Severe deformity of the femoral neck	Avascular necrosis, arthritis, arthrodesis

### 2.6. Statistical analysis

Descriptive statistics were used for continuous variables, and the results of statistical analysis were presented as mean and standard deviation (SD); the total number (n) and percentage (%) were used to express discrete variables. All variables were analyzed with SPSS 20.0 (SPSS, IBM, United States).

## 3. Results

A total of 19 patients aged between 12 and 19 years, all operated with transphyseal fixation using combined cannulated cancellous screw and Kirschner wire, were included and retrospectively studied; the results are presented as follows:

There was a male predominance in this cohort (76% cases); the mean age was 15.37 ± 2.23 years; the most frequent mechanism of injury was fall after sport activities in school (the height jump), followed by road traffic accident: 66% and 34%, respectively. The right side was frequently injured (57% of cases) and Delbet type III was the most encountered (54%). The mean intraoperative time was 54.71 ± 7.85 min, length of hospital stay (LOS) was 8.34 ± 1.81 days, and time to surgery was 2.60 ± 1.16 days (Table 2).

**Table 2:** Patients’ demographics and operation parameters.

Variables	Values	Percentage
Age (mean ± SD), years	15.37 ± 2.23	—
Operation time (mean ± SD), min	54.71 ± 7.85	—
BMI (kg/m^2^)	21.1 ± 3	—
Hospital length of stay (mean ± SD), days	8.34 ± 1.81	—
Time to surgery (mean ± SD), days	2.60 ± 1.16	—
Gender, *n* (%)		
Male	14	74
Female	5	26
Mechanism of injury, *n* (%)		
Road traffic accident	6	34
Sport injury (high jump, etc.)	13	66
Side of injury, *n* (%)		
Right	11	57
Left	8	43
Delbet classification		
Type I	0	—
Type II	6	29
Type III	10	54
Type IV	3	17

Radiographic healing was observed after 3.31 ± 1.04 months; coxa vara was encountered in 2 (11%) cases, osteoarthritis in 1 (5%) case, and spontaneous dislocation in 1 (5%) case. The mean ROM was clinically acceptable. Nonunion, femoral neck AVN, and aseptic screw loosening were not encountered in any case of the studied patients. The assessment of femoral neck shortening revealed a moderate shortening in 16% of cases and no case of severe shortening. Clinical assessment with the Ratliff system showed a good result in 89% of patients and a fair result in 11% of patients (Table 3).

**Table 3:** Postoperative follow-up and complications.

Variables	Values	Percentages
Follow-up time (mean ± SD), years	5.83 ± 3.76	—
Fracture healing time (mean ± SD), months	3.31 ± 1.04	—
Nonunion *n* (%)	0	—
Coxa vara angle (degree), *n* (%)	2	11
Osteoarthritis, *n* (%)	1	5
Femoral neck avascular necrosis *n* (%)	0	—
Aseptic Screw loosening, *n* (%)	0	—
Spontaneous dislocation	1	5
Hip range of motion ROM (degree)		
Abduction	30.1 ± 9	—
Adduction	10 ± 5.6	—
Flexion	105 ± 6.9	—
Femoral neck shortening, *n* (%)		
<0.5 cm	16	84
0.5–1 cm	3	16
>1 cm	0	—
Clinical assessment (Ratliff’s system)		
Good	17	89
Fair	2	11
Poor	0	

ROM, range of motion.

## 4. Discussion

The treatment approach of femoral neck fracture in pediatric patients is controversial; the current available literature recommend various guidelines such as conservative management with prolong balanced traction, prolong casting, non-weight-bearing period with the use of crutches, percutaneous fixation, and internal fixation using cannulated screw, fixation with K-wires, or Moore's pins (1, 4, 5, 8, 9, 13, 14, 20–24). Given the vulnerability of blood vessels of the femoral neck in pediatric patients, there have been several studies reporting on post-therapeutic complications, especially avascular necrosis. We designed our proper management approach consisting in transphyseal fixation using combined CCS with Kirschner wire with the intention of reducing the rate of complications and fixation failure in adolescent hip fracture.

Some young patients with femoral neck fractures are close to adults in height and weight, and in the case of patent epiphysis, we chose cannulated screw fixation without crossing the epiphysis; additionally, it is necessary to increase the stability of the implant by inserting Kirschner wires into its inner holes, while reducing the impact of implants on the epiphysis; moreover, for fracture of femoral neck in adults, the standard treatment option is a 7.3-mm diameter cannulated screw. The best choice is a real 2.5 mm Kirschner wire, which is just enough to pass through the hollow screw bore. So we believed that we could use the Kirschner wire in conjunction with our hollow screw to provide the best and strongest biomechanical fixation. In contrast, patients with closed femoral physis are considered to have sufficient bone maturity and the protocol of adult femoral neck fracture management principle is applied; we can safely advance screws further within the femoral neck without the need of Kirschner wires.

The choice of internal fixation through the epiphysis, whether it is a cannulated screw or a dynamic hip screw, can theoretically provide better biomechanical stability, but whether it affects the development of the proximal femoral epiphysis or not is inconclusive. Results of trials and long-term follow-up may provide evidence for better treatment options.

Fracture classification is an important tool for the management of fracture in any population group. In pediatric patients, Patterson et al. (9) reported that the most common type of femoral neck fractures are Delbet type II and type III fractures; moreover, authors detailed that these are often displaced and the choice of transphyseal cannulated screws is indicated in those aged ≥10 years. In the current study, consistent findings were reported with the fracture types; the patients’ ages varied from 12 to 19 years and were all treated with cannulated screws (cross-epiphyseal fixation). The particularity in our study was that the guide wire (Kirschner wire) used during surgery were left in place after surgery and were removed along with the cannulated screw during screw removal after fracture healing. We believed that adding a guide wire within the cannulated screw will strengthen stability of the internal fixation; moreover, we also believed in vulnerability of the femoral head cartilage which needs to be protected from any damage (exposing to the postoperative osteoarthritis). Therefore, we often avoid the screw reaching underneath the femoral head cartilage by choosing shorter screw, and a Kirschner wire of longer medial end (longer than the CCS) is used to compensate the length of the screw (Figure 1D). According to the principle of femoral head screw fixation, distance between the articular cartilage of the head and screw must be 0.5 cm on anteroposterior radiographic view; this has to be respected and compensated by the Kirschner wire. We have not found a consistent similar management design in the current literature. The outcome according to the Ratliff assessment system revealed a good result in 89% of cases.

AVN is one of the most serious complications of femoral neck fracture in children; it is a result of damage to the blood supply of the femoral head caused by fracture displacement during the time of initial trauma and tamponade effect of the hip joint. A rise in intracapsular pressure brought on by hemarthrosis (posttraumatic hemarthrosis) due to the tamponade effect has been associated with avascular necrosis after femoral neck fractures. Intramedullary and synovial venous drainage are disrupted by a femoral neck fracture, which also interferes with intracapsular fluid drainage and increases intracapsular hypertension. The most recent research supports surgical intraoperative drainage of intracapsular hematoma. Evacuation of the intracapsular hematoma has been recommended, especially in nondisplaced fractures, since it may lessen intracapsular pressure and enhance blood flow to the femoral head (25–27). According to some authors, routine hemarthrosis aspiration is not recommended because the amount of aspirated hemarthrosis is typically fairly small and intracapsular pressure readings in the antalgic position are below the diastolic blood pressure (28). In a study conducted by Maruenda et al (29), hip traction in the antalgic position led to a greater reduction in intracapsular pressure than hemarthrosis aspiration in both displaced and nondisplaced fractures. In the current investigation, closed reduction was favored over intraoperative drainage. We believed that closed reduction was beneficial to the patient. In the course of perioperative management, as a routine for all patients with femoral neck fracture, preoperative skeletal traction was given (3–4 kg) to reduce the intracapsular pressure.

The incidence of AVN is closely related to the type of fracture according to the Delbet classification and time to surgery (7). Controversial data on the incidence of postoperative AVN following pediatric femoral neck fracture are available in the literature. Some authors reported that the range of osteonecrosis of the pediatric femoral neck varies from 0% to 92% (30), but it was reported to be 28.58% by one another author (6). Other authors linked the incidence of AVN to the fracture type. According to this, one scholar reported that the rate AVN is 38%, 28%, 18%, and 5% in type I, II, III, and IV, respectively according to the Delbet classification (7). Riley et al. (30) reported an osteonecrosis rate secondary to femoral neck fracture in the pediatric population for Delbet type I to IV fractures to be 50%, 28%, 8%, and 10%, respectively.

On the other hand, some authors associated a higher incidence of AVN to the patient's age, given that this incidence is higher in patients aged above 12 years (6, 13). Riley et al. (30) reported 20% of osteonecrosis in pediatric femoral neck fractures and presented age (≥11 years old) as the only predisposing factor. Singh et al. (14) found that there is a high incidence of AVN in patients aged 10 years or older with Delbet type II fracture. In our study, this age factor could not be verified based on our dataset, as we only had 19 patients, which was not enough to comment on the rate of AVN, and all of them were 12–19 years.

The timing of the operation should also be taken into consideration when discussing the predisposing factor for avascular necrosis. Unfortunately, most doctors do not place enough emphasis on pediatric femoral neck fractures. Furthermore, the history of presenting the complaint revealed that, in most patients in our series, patients’ parents or relatives usually reported pain around the hip as “simple muscle sprain, and often think the pain can resolve after few hours”; in fact, people frequently admit that it is the endurance of discomfort beyond more than 24 h that drives them to seek medical attention. As a result, patients frequently arrive late at the hospital; this delay in arrival at the hospital was previously noted by other authors in the literature (31). These are significant variables in the delay in surgical intervention. In our study, the time to surgery was 2.60 ± 1.16 days; despite the good results, the current study does not rule out the possibility that the timing of the procedure plays a role in avascular necrosis.

The presence of arthritis concomitant with AVN had already been reported; After a long-term follow-up period on 38 patients who received treatment with internal fixation following femoral neck fracture, arthritic changes were found in 18 (34%) patients; the authors reported that there was a strong correlation between the occurrence of avascular necrosis and arthritic change (8), but whether the arthritis was related to the fracture type or pattern of the fracture was not clearly understood. Another literature study reported that, coxa vara is a known risk factor for hip arthritis (11). In the current study, only 1 (5%) patient developed osteoarthritis and no case of avascular necrosis was noticed; this was not consistent to with the above finding.

The time to operation probably influenced the incidence of occurrence of complication, notably the AVN. Dendane et al. (6) reported an increased incidence of avascular necrosis in patients operated after 48 h. Yeranosian et al. (11) reported a delay of the surgical intervention of more than 24 h as one of the contributing factor for higher incidence of AVN.

In the current study, all our patients were operated as early as possible (2.60 ± 1.16 days). We did not have any case of AVN, but a direct connection between the times to the operation and the absence of femoral head AVN was not clearly understood. However, we still believed that the quality and stability of the internal fixation played an important role.

According to some authors, Delbet type I, II, and III intracapsular fractures are exposed to synovial fluid, which interferes with the fracture healing (11). In our study, most of our patients (83%) had Delbet type II (29%) and type III (54%) intracapsular fractures and the average healing time was 3.31 ± 1.04 months, which was clinically acceptable; however, the connection between the fracture type/location and fracture healing time could not be clearly established, and we acknowledge that, based on our limited number of cases, we could not reach an objective conclusion.

The neck–shaft angular deformity has also been previously commented. Wang et al. (13) reported a mean neck–shaft angle (NSA) of 138.3° ± 10.44° on the affected side compared to 139.55° ± 10.19° on the non-affected side of the femoral neck; the authors mentioned that this angle was comparable to the normal hip. Moon and Mehlman (7) defined coxa vara as neck–shaft angles of <130°. In a study conducted in pediatric patients aged between 3 and 16 years, treated with internal fixation using cannulated screw, Morsy (8) reported an incidence of Coxa vara of 36%. Yaokreh et al. (15) reported a rate of 54.5% patients who developed coxa vara, with a mean neck–shaft angle of 102.16° ± 12.07°, ranging from 90°–118°, all present in the group of patients treated with conservative management. The anatomical reduction and internal fixation can be used to efficiently control the incidence rate of coxa vara (7, 14). In our study, all patients were treated surgically with internal fixation and coxa vara was observed in two (8%) patients; coxa vara could be easily tolerated (105° and 110° in two patients) as those patients had no symptoms and had good Harris Hip Score (HHS). this finding was consistent with the literature (10). However, it was unclear whether the cross-epiphyseal fixation had reduced the risk of coxa vara incidence.

Femoral neck shortening is also a common finding in patients with femoral neck fracture. Dendane et al. (6) reported 4 cases of full leg shortening (with the shortening ranging from 2 to 4 cm) out of a series of 21 cases, all associated with coxa vara; they supported that architectural changes to proximal end of the femur including coxa vara, cervicocephalic necrosis, improper reduction, and/or fixation are usually associated with leg shortening. Moon and Mehlman (7) reported 4 cases of leg-length discrepancy out of 25 cases; leg shortening was associated with the type of fracture (Delbet type II, III, and IV) and other complications such as coxa valga, trochanteric overgrowth, AVN, and coxa vara. Leg shortening reported by the authors were all less than 5 cm (1–3 cm). Morsy (8) reported shortening in 55% patients with a shortening rage of 1–5 cm; the author mentioned that this shortening could be attributed in majority of cases to coxa vara, AVN, and premature physeal closure. In this study, 84% of cases had a shortening of less than 0.5 cm and 16% had a shortening of 0.5–1 cm.

The fixation failures including screw loosening and screw breakage are also reported. In a study conducted by Sun et al. (32), there were 56.0% cases of lateral withdrawal of screws in the group of patients treated with partially threaded screws compared to 21.3% in those treated using fully threaded screws; in both cases, the failure was lateral withdrawal of the screw. According to the authors, the difference in both groups was probably related to the choice of fixation device. Estrada et al. (33) reported that in older patients, the bone quality plays an extremely important role in the failure or success of internal fixation outcome. In the present study, our patients were usually treated with partially threaded screws and we observed 0% fixation failure. Moreover, the patient group in our study had a different specificity which is their age (all 19 years and below) with good bone quality. In contrast, the screws in the majority of our patients were buried within the cortex of the bone.

Other complications such as nonunion, neck collapse, and delayed union are also common in fracture of the femoral neck. Yeranosian et al. (11) reported an 8.4% nonunion rate independent of the treatment method. Another scholar reported a 36% nonunion rate (8). The absence of signs of fracture healing at 6 months (ranging from 3 to 12 months) from injury is known as nonunion. Referring to the definition given by the Food and Drug Administration (FDA) in the United States, a period of 6 months is needed to consider a nonunion plus 3 additional months to verify that nonunion is established (22, 34). In the current series, there was no case of nonunion and all fractures healed at 3.31 ± 1.04 months. This finding was consistent with the report available in literature (15).

**Figure 2:**
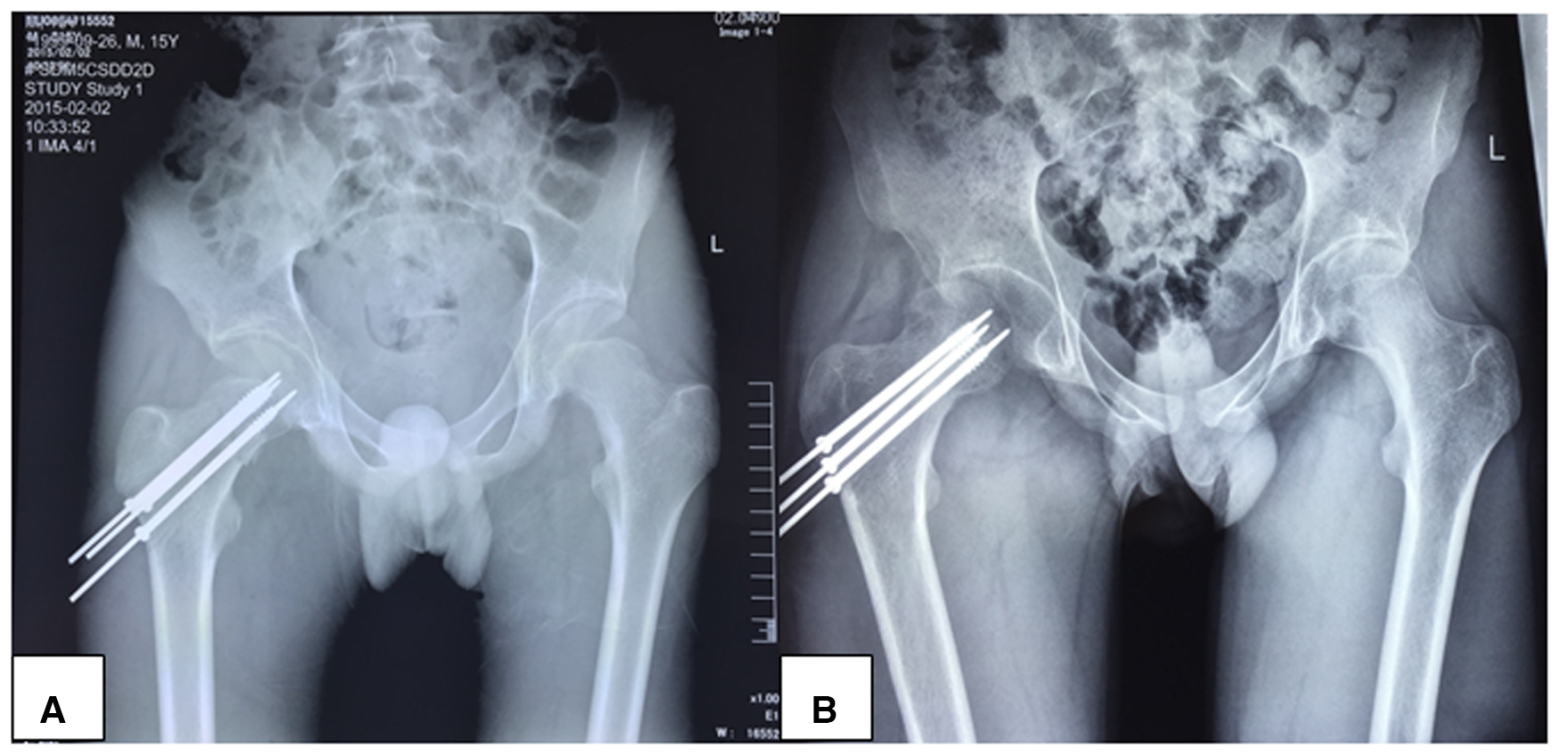
A 15-year-old male patient who sustained a Delbet type II right hip fracture after a fall from height treated operatively with transphyseal fixation with cannulated screw combined with K-wire (**A**); the patient was readmitted 10 months later and diagnosed with spontaneous dislocation (**B**).

We did not register any case of neck collapse among the patients enrolled in our dataset; however, we reported a case of spontaneous dislocation in a noncompliant patient, a 15-year-old male (who sustained a Delbet type II femoral neck fracture and operated with our protocol approach) 10 months postoperatively (Figures 2A,B). This dislocation was immediately repaired manually under general anesthesia. Unfortunately, we could not establish whether this event was related to the fixation protocol or not, rather we associated it to the early stress exercises applied to the affected hip by the patient.

This study presents some limitations: first, its retrospective nature makes it susceptible to possible minor errors eventually because the patients were selected and not randomly assigned. Second is the lack of consistent available studies using the same treatment protocol with which we could confront our findings; the presence of some confounding variables such as patients’ age, BMI, and fracture type are potentially susceptible to affect the clinical outcome and findings. Finally, this series had a limited sample size and we are aware that this small sample size probably influenced our outcome. Due to the rarity of such a fracture (1%), we could not collect an important number of patients despite the number of years on which we extended our study and data collection.

We, therefore, acknowledge that more comparative studies and a randomized control trial are needed to further authenticate our findings.

## 5. Conclusion

In conclusion, in our series, the use of cross-epiphyseal fixation using the combined cannulated cancellous screw with Kirschner wire approach in adolescent femoral neck fractures demonstrated viable outcomes. We, therefore, present this as an alternative for the management of adolescent femoral neck fractures with patent physis.

## Data Availability

The original contributions presented in the study are included in the article/supplementary materials, further inquiries can be directed to the corresponding author/s.
